# Supplementary material for: Human and nonhuman primate meninges harbor lymphatic vessels that can be visualized noninvasively by MRI
Source: eLife. 2017 Oct 3;6:e29738. doi: 10.7554/eLife.29738 (PMC5626482; doi:10.7554/eLife.29738)
Supplement: Figure 3—source data 1. [file elife-29738-fig3-data1.docx]

**Table 1. Human tissue sampling**

| Patients | Demographics | Main diagnosis | Tissue sampling |
| --- | --- | --- | --- |
| #1 | 60 years old  at death, female | - Progressive multiple sclerosis - Natalizumab-associated progressive multifocal leukoencephalopathy | Autopsy   - 1 coronal section of the dura, including   the superior sagittal sinus   - 1 transversal section of the dura, including the superior sagittal sinus - 1 coronal section of the dura, including   the straight sinus   - 2 blocks of the dura laterally the convexity - 3 blocks of the skin (positive control for lymphatic markers) |
| #2 | 77 years old at death, male | - Progressive multiple sclerosis - Pneumonia | Autopsy   - 1 coronal section of the dura, including the superior sagittal sinus |
| #3 | 33 years old at surgery, male | Refractory epilepsy | Anterior temporal lobectomy |
