# Supplementary material for: Human and nonhuman primate meninges harbor lymphatic vessels that can be visualized noninvasively by MRI
Source: eLife. 2017 Oct 3;6:e29738. doi: 10.7554/eLife.29738 (PMC5626482; doi:10.7554/eLife.29738)
Supplement: Figure 4—source data 1. [file elife-29738-fig4-data1.docx]

**Table 2. Marmoset tissue sampling**

| Animals | Demographics | Main procedures | Tissue sampling |
| --- | --- | --- | --- |
| #1 | 4.4 years old  at death, male | - Baseline MRI - EAE induction - MRI follow-up scans | - MRI-matched coronal sections of the brain, including the dura mater - 3 blocks of the skin (positive control for lymphatic markers) |
| #2 | 10.3 years old  at death, male | - Baseline MRI - Stroke under general anesthesia for tuberculosis blood tests | - MRI-matched coronal sections of the brain, including the dura mater |
| #3 | 3.7 years old  at death, male | - Baseline MRI - EAE-induction - MRI follow-up scans | - Whole-mount of the dura mater |
